# Terrain Point Cloud Assisted GB-InSAR Slope and Pavement Deformation Differentiate Method in an Open-Pit Mine

**DOI:** 10.3390/s20082337

**Published:** 2020-04-20

**Authors:** Xiangtian Zheng, Xiufeng He, Xiaolin Yang, Haitao Ma, Zhengxing Yu, Guiwen Ren, Jiang Li, Hao Zhang, Jinsong Zhang

**Affiliations:** 1 School of Earth Science and Engineering, Hohai University, Nanjing 211100, China; zhengxt@hhu.edu.cn; 2 China Academy of Safety Science and Technology, Beijing 100012, China; yangxl@chinasafety.ac.cn (X.Y.); maht@chinasafety.ac.cn (H.M.); yuzx@chinasafety.ac.cn (Z.Y.); rengw@chinasafety.ac.cn (G.R.); lijiang@chinasafety.ac.cn (J.L.); zhstrive@126.com (H.Z.); 3 Beijing Institute of Surveying and Mapping, Beijing 100038, China; 13264361838@163.com

**Keywords:** ground-based synthetic aperture radar interferometry (GB-InSAR), deformation monitoring, geometric and scattering model, slope deformation monitoring

## Abstract

Ground-based synthetic aperture radar interferometry (GB-InSAR) is a valuable tool for deformation monitoring. The 2D interferograms obtained by GB-InSAR can be integrated with a 3D terrain model to visually and accurately locate deformed areas. The process has been preliminarily realized by geometric mapping assisted by terrestrial laser scanning (TLS). However, due to the line-of-sight (LOS) deformation monitoring, shadow and layover often occur in topographically rugged areas, which makes it difficult to distinguish the deformed points on the slope between the ones on the pavement. The extant resampling and interpolation method, which is designed for solving the scale difference between the point cloud and radar pixels, does not consider the local scattering characteristics difference of slope. The scattering difference information of road surface and slope surface in the terrain model is deeply weakened. We propose a differentiated method with integrated GB-InSAR and terrain surface point cloud. Local geometric and scattering characteristics of the slope were extracted, which account for pavement and slope differentiating. The geometric model is based on a GB-InSAR system with linear repeated-pass and the topographic point cloud relative observation geometry. The scattering model is based on k-nearest neighbor (KNN) points in small patches varies as radar micro-wave incident angle changes. Simulation and a field experiment were conducted in an open-pit mine. The results show that the proposed method effectively distinguishes pavement and slope surface deformation and the abnormal area boundary is partially relieved.

## 1. Introduction

Deformation monitoring can reveal unstable slope surfaces to prevent serious safety hazards. It is deployed to determine changes in the shape and displacements of a deformable body in temporal and spatial domains [1]. Advancements in signal processing, integrated circuit, microwave, and antenna technologies have made synthetic aperture radar (SAR) a notable innovation in the radar technology field. Interferometry is a technique for extracting deformation information by comparing the observed phases before and after the deformation of an object. SAR interferometry technology can be used for all-weather situations and the high-precision monitoring of ground surface deformations across large areas [2]. Ground-based synthetic aperture radar interferometry (GB-InSAR) has been tested in various fields in recent years including the monitoring of landslides, open-pit mines, ground subsidence, architectural structures, bridges, sinkholes, and glacier displacements [3,4,5,6,7,8]. Despite these achievements, there are yet obstacles hindering the practical application of GB-InSAR deformation monitoring. Traditional SAR can obtain 2D images in steep and complex terrain, but 3D objects may be severe distorted on the 2D image which obscures several targets [9,10,11]. SAR sensors can resolve different monitored targets based on their slant ranges from the targets to the radar center and the angles away from the centerline of the radar antenna middle beam. Unlike the orthographic projection of a traditional topographic map and the central projection of photogrammetry, SAR-based image projection generally requires manual interpretation to identify a deformed target and its position in the planar coordinate system of a radar image. The interpretation of radar images requires understanding and judgment of radar observation geometry and imaging principles, matching with 3D scenes, and accurately locating deformation areas and variations. The analyst must have sufficient knowledge to ensure appropriate such identifications. The issue of accurately interpreting 3D measurement results is key regarding successful GB-InSAR implementation. 

Previous research has explored GB-InSAR mapping technology in the GB-InSAR geometric mapping context. Tapet et al., for example, integrated terrestrial laser scanning (TLS) and GB-InSAR to monitor heritage building deformations and accurately located deformed areas [5]. Yue et al. fused GB-radar and survey robot data to determine the spatial relationship between the robot monitored 3D deformations and the GB-radar coordinate system [12]. The method was used for 1D deformation and was not expanded in high dimensions. GB-InSAR and InSAR have unique observation geometry and measurement modes, so applying the InSAR processing method to GB-InSAR produces a substantial error. The integration of high-precision external auxiliary measurements may be an effective solution to this problem. Zou, of Wuhan University, fused ground-based SAR and 3D laser scanning data for the 3D visualization of GB-InSAR in a hydro-dam deformation monitoring experiment using point cloud, coordinate transformation and projection, and 2D four-parameter transformation techniques [13]. Yang et al. proposed a geometric 3D matching method based on both range and azimuth constraints for frontal and lateral strip SAR and terrain data, [14]. Wang et al. used 3D laser scanning data to assist the 3D coordinate transformation of GB-InSAR image pixels; they also applied a scaling factor method based on the parameter estimation of a similar transformation. They put forward the idea of mismatching correction based on control points [9]. In general, inconsistencies among systems, variations in the measurement environment, and scene image changes remain problematic. In an experiment on ground-based SAR deformation monitoring, Yang et al. used a positioning method to determine the rail endpoint coordinate and artificial targets [4]. Lombardi et al. [15] integrated GB-InSAR and 3D laser scanning for landslide monitoring. Zheng et al. investigated data fusion and deformation comparison with GB-InSAR and 3D laser scanning in a controlled field test and later they integrated GB-InSAR, TLS and unmanned aerial vehicle photogrammetry (UAV) in a rock slide residual mass emergency monitoring case [1,16].

The geometric mapping of the GB-InSAR image and 3D terrain data is an important step in the deformation monitoring process. During a monitoring case in an open-pit mine, we encountered a problem: mapping results cannot differentiate slope steps differences. The problem confused geo-engineers in analyzing the deduction of spatial and temporal deformation patterns. Based on the above analysis, we can conclude that extant geometric mapping methods are deficient in the mapping relation of multiple spatially distributed target points of the radar pixel with the point cloud, as well as the 3D features (e.g., structure, texture, and occlusion) in reflecting targets. The points cloud may help inversion of the scattering difference of GB-InSAR, which would help differentiate the pavement and slope. Conventional mapping methods based on nearest-neighbor interpolation cannot be effectively applied to SAR pixels. Based on the basic cognition that SAR image is a kind of target scattering characteristic difference representation map. We develop a method that is comprised of a geometric mapping and scattering variance weighting model. The model is introduced in setting the interpolation weight of points in one radar pixel. The method may help in identifying shadows and layover in topographically rugged areas.

The paper is organized as follows. Section 2 discusses the problem we encountered and solving scheme by an improved linear GB-InSAR and TLS data fusion method. Section 3 further discusses the GB-InSAR system and the rough surface slope topographic data model. The GB-InSAR image and spatial point cloud 3D mapping method, which is based on geometric and scattering models, is also delineated in Section 3. Section 4 presents a data fusion experiment in an open-pit mine in China, which was conducted to validate the model. Section 5 discusses the influence factors and possible solutions. Section 6 provides a summary and recommendations for future research.

## 2. Linear GB-InSAR and TLS Data Fusion

### 2.1. Problem and Solving Scheme

**Figure 1:**
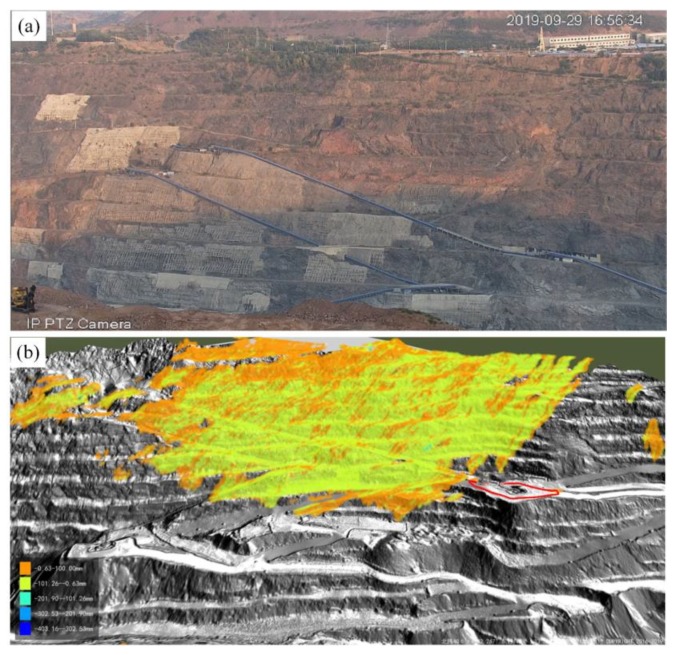
(**a**) Optical image of the target open pit slope. (**b**) It is difficult to distinguish the pavement surface from the slope surface using the mapping result generated by nearest-neighbor interpolation. The orange color areas consist of several pixels across multiple slope steps. A ground-based synthetic aperture radar interferometry (GB-InSAR) cumulative deformation map was produced in a month, and was mapped on the terrain model. The orange-colored area shows great deformation but is difficult to interpret by geotechnical engineers.

A time-series of 2D images was fused with GB-InSAR and the 3D terrain surface point cloud data with TLS as shown in Figure 1. This fusion is a step-wise process. The key processes are as follow:

- Calculate relative ranges and azimuth angle according to GB-InSAR monitoring geometry.
- Nearest-neighbor interpolation for one radar pixel-to-multiple terrain surface points mapping.
- 3D visualization.

Figure 1b shows the mapped result, where the gray-scale color marks the terrain model generated by the TLS point clouds, which consists of several 1 m^2^ patches. The colored layer is the GB-InSAR deformation 3D projection “cloud diagram”. However, the mapped result shows no differences in the pavement and slope. Geotechnical experts cannot intuitively evaluate the stability of a given slope according to the "cloud diagram" of deformation changes, and the results are shown as a band-like pattern. These bands do not conform to any constitutive model of slope deformation and would not help geotechnical destroy deduction. Additionally, the open pit examined here was excavated in strict accordance with the engineering design. There is a resting angle in the monitored slope; the safety factor is higher when the slope and road surface are at an angle. The result cannot verify the safety angle, so the mapping process should be improved. 

**Figure 2:**
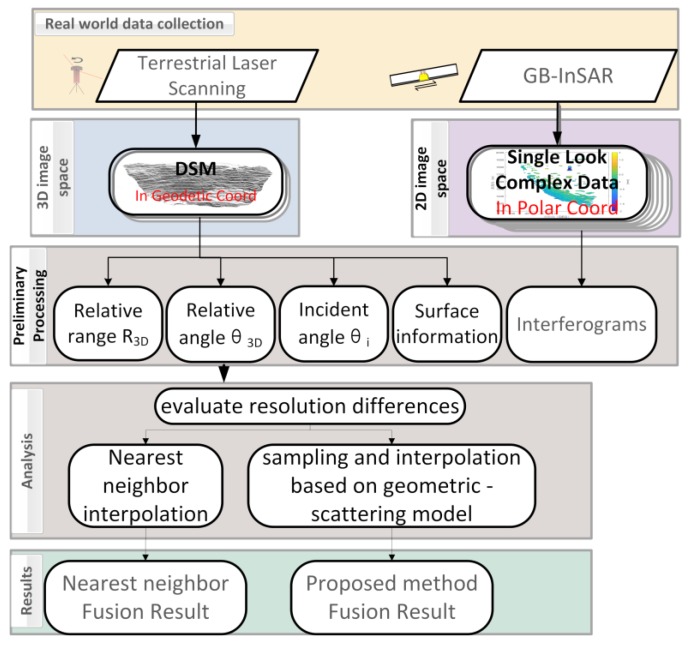
Logical experiment scheme of the data fusion between GB-InSAR interferograms and terrain point cloud data using nearest-neighbor interpolation and geometric-scattering model separately.

The study flow of the paper is as shown in Figure 2. It depends on the assumption that the existing nearest-neighbor interpolation does not consider the incident angle and surface information. We further research to provide a solution to this problem and add the two factors in our geometric and scattering model. 

### 2.2. GB-InSAR and Point Cloud

#### 2.2.1. GB-InSAR Images

**Figure 3:**
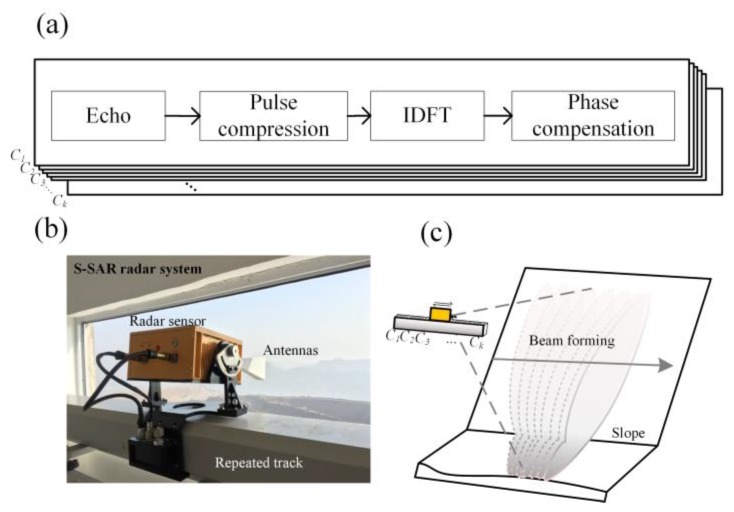
The synthetic aperture model was observed at a fixed-station, overhead perspective. (**a**) The key process of GB-InSAR echo data processing. (**b**) S-SAR radar system which contains transmitting and receiving antennas, radar sensor and a linear rail. (**c**) Ideal state: "One-step, one-stop" SFCW signal transmitting and receiving mode of straight-line repeated-track controlled by a pulse-width modulation (PWM).

The proposed method was validated in an experiment at an open-pit mine in China using a Ku-band GB-InSAR system developed by the China Academy of Safety Science and Technology (CASSAT) and named as slope SAR (S-SAR). The system is shown in Figure 3.

The radar sensor gathered data based on the principle of independent propagation and superposition of electromagnetic waves. The data were coherent after processing. A synthetic-aperture radar is generally an antenna array of N numbers of antennas. The echo signals were received by each antenna and then coherently superimposed. The common GB-InSAR may use an stepped frequency continuous wave (SFCW) signal or linear frequency modulation continuous wave (LFMCW) signal. The SFCW system is easier than the LFMCW to implement, hardware-wise. The linear rail driver adopts the pulse-width modulation (PWM) signal to trigger and control the sliding platform of the linear track "step-by-step and stop". In a single scan from start to end, the original data as-obtained were processed and saved in a complex array. Each element $C_{1},C_{2},\cdots ,C_{n}$ in the array corresponds to a specific location on the rail, allowing the data of a certain frequency band to be obtained. The term “repeat-track”, here, refers to passing the same area repeatedly. In this approach, the system acquired SAR data sets by passing the same area twice, covering it with a slightly different viewing geometry. The imaging geometry of the repeat-track SAR interferometry is shown in Figure 3c. On the first pass, the radar wave was transmitted from the transmitting antenna every 1 s. After interaction with the terrain, the backscattered return was also recorded by receiving antenna every 1 s. The signal was then processed to a complex SAR image, with 2 s on the second pass; the signal was processed to another complex SAR image. The system made repeated observations of specific scenes at different time points to obtain radar images in a time series. The phase changes of the image sequence can be analyzed to obtain high-precision deformation information of the observed scene. The elevation in the monitored scene fluctuates as 2D images were gathered in 3D space. The GB-InSAR used an overhead perspective to monitor unstable slopes. 

**Figure 4:**
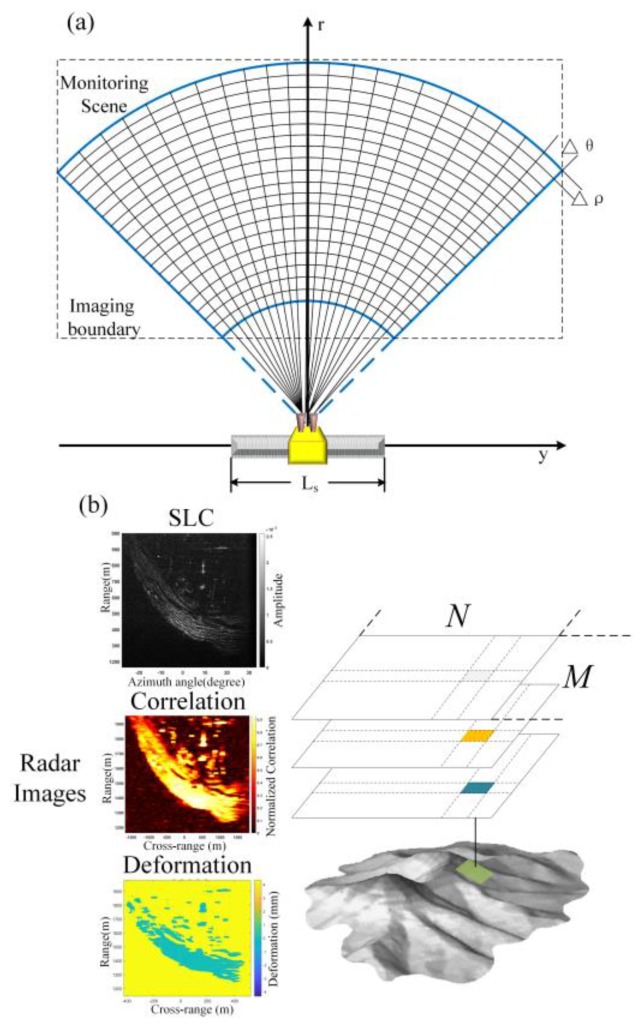
(**a**) The GB-InSAR image grid. The spatial resolution of the grid is lower than terrestrial laser scanning (TLS) points cloud resolution. Each grid may correspond to multiple terrain surface model points. (**b**) GB-InSAR image pixel responds to the same slope area after 2D image co-registration.

Figure 4 shows the characteristics of the smallest element in a radar image, which is the key parameter in the matching process. The GB-InSAR image grid is the projected image of the observed scene on the slant range plane. After the image co-registration of the GB-InSAR and single look complex image (SLC) in the precise orbit mode, images with the same (M, N) indexed pixel, coherence, and deformation characteristics continuously correspond to the same side of the target slope in the ideal state. In monitoring unstable slopes, the local area observed by GB-InSAR usually has its own 3D coordinate system as well as the topographic data obtained by TLS, total stations, GPS, or interferometric SAR. The TLS is fast and accurate; its monitoring scope is similar to that of the GB-InSAR. (Rigel’s vz4000 model has a mapping range of 4000 m, while the VZ6000 can reach 6000 m in a better environment [17]). In most cases, the digital surface model (DSM) can be rapidly constructed via TLS. The digital elevation model (DEM) was generated after elevation correction. Automatic deformation monitoring with GB-InSAR works via 2D grid imaging methods, which are stable, rapid, and well-tested. This section focuses on the preprocessing of the topographic point cloud as 2D polar coordinate information. This stepwise process included point cloud preprocessing followed by the acquisition of the distance and perspective of spatial points relative to the GB-InSAR. 

#### 2.2.2. Point Cloud

**Figure 5:**
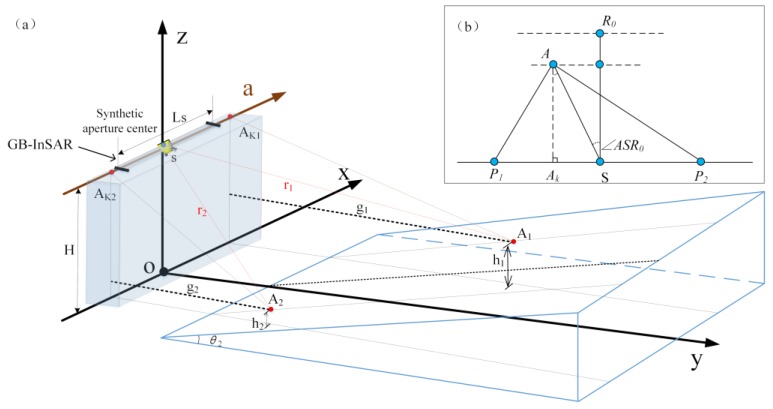
(**a**) The geometric mapping relationship between normal center rectangular coordinate system GB-InSAR and 3D terrain data. (**b**) Terrain point cloud azimuth angle top view.

As shown in Figure 5a, the distributed ideal targets were selected as the reference points $A_{1}(x_{1},y_{1},z_{1})$ and $A_{2}(x_{2},y_{2},z_{2})$ on the 3D terrain slope surface located on both sides of the central axis of the synthetic aperture.

(1) $$\left\{ \begin{array}{l} r_{1}=\sqrt{{\left(x_{1}-x_{s}\right)}^{2}+{\left(y_{1}-y_{s}\right)}^{2}+{\left(z_{1}-z_{s}\right)}^{2}}\\ \;\;={\left\|S-A_{1}\right\|}_{2}\\ r_{2}=\sqrt{{\left(x_{2}-x_{s}\right)}^{2}+{\left(y_{2}-y_{s}\right)}^{2}+{\left(z_{2}-z_{s}\right)}^{2}}\\ \;\;={\left\|S-A_{2}\right\|}_{2} \end{array}\right.$$

where ${\left\|\;\cdot \;\right\|}_{2}$ represents the Euclidean 2-norm of the space vector. As shown in Figure 5b, the local azimuth angle in the 2D plane coordinate system can be calculated as follows:

(2) $$∠R_{0}SA=\arccos(\frac{\left|A_{k}A\right|}{\left|A_{k}S\right|}),$$

where $R_{0}$ represents the center of the monitoring area, $∠R_{0}SA$ is shown in Figure 5b which denotes for relative view angle of target A. The angle needs a further conversion according to the specified radar image coordinates when geometric mapping. 

## 3. Methods

### 3.1. Geometric Mapping Between Image Space and Terrain Space

Let I denote for the deformation map 2D matrix. The size of I is (M,N) and is coded in polar coordinates (r,θ) or pseudo-polar coordinates (r,sin(θ)). Let $I_{pcl}$ represent the 2D matrix formed by the relative range and relative azimuth angle derived by Equations (1) and (2). There is no existing deformation value corresponding to the internal coordinates of $I_{pcl}$ in I. Then, the mapping relation we have to calculate is $f:I\to I_{pcl}$. In our case, the spatial resolution of the point cloud is higher than that of radar, the appropriate method of resampling and interpolation is needed to assign deformation value to the point cloud. Mapping relations are as follows:

(3) $$\{\begin{array}{l} \begin{array}{l} f1:(r,\sin(\theta ))\to (m,n)\\ f2:(r,\theta )\to (X_{1},Y_{1},Z_{1})\\ f3:(X_{1},Y_{1},Z_{1})\to (X_{t},Y_{t},Z_{t}) \end{array}\\ f=f1\circ f2\circ f3 \end{array}$$

where f1 represents the mapping of pseudo-polar coordinates (e.g., plane coordinates) to pixel coordinates, (m,n) represents the 2D image pixel index. f2 is the mapping of polar coordinates (e.g., plane coordinates) to the local coordinate system of the point cloud, and $\left(X_{1},Y_{1},Z_{1}\right)$ denotes local coordinates. f3 is the mapping of the local coordinate system of the point cloud to the geodetic coordinate system, $\left(X_{t},Y_{t},Z_{t}\right)$ stands for the geodetic coordinates and symbol ‘∘’ represents a cascade. 

### 3.2. Geometric and Scattering Weight Model

**Figure 6:**
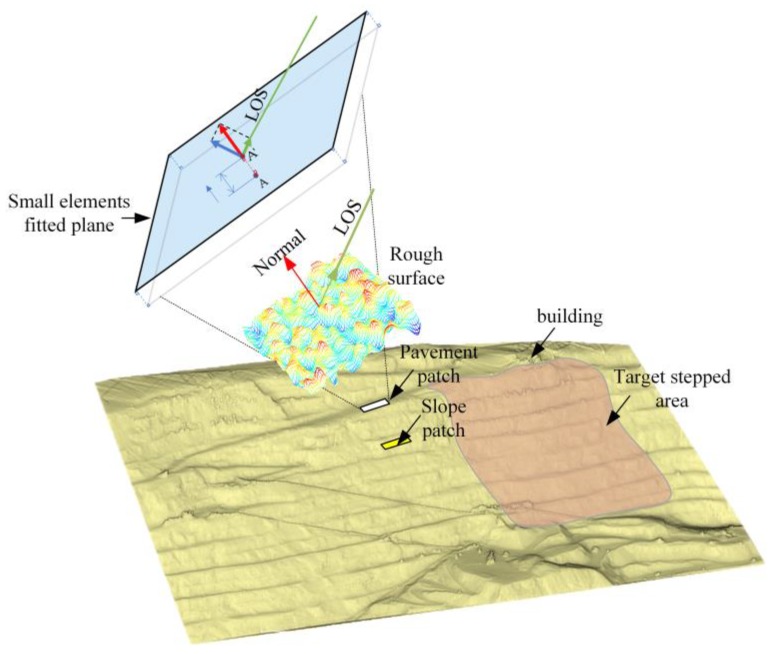
Terrain point cloud introduces greater detail so that radar wave incident angles define geometric and scattering models. The model helps in solving the one-to-many problem during the data fusion process. For the road surface and slope surface of the slope, they have completely different relative incident angles.

As shown in Figure 6, slope surface nodes and their nearest neighbor discrete points in the DEM consist of small surface element patches. Traditional matching methods mostly work based on the geometric model of the radar and terrain. Here, a geometric and scattering model was applied for the patches to solve the one-to-many problem. 

The scattering model is commonly used in SAR image simulations. The problem to be solved, as discussed in this section, is similar to that of SAR image simulation: The terrain point cloud data imported externally have higher dimensions and resolution than the radar data. Deformation results with high dimensionality and resolution can be retrieved based on the existing GB-InSAR monitoring results. The most commonly used scattering model is the small surface element model [18], each small surface element is tangent to the actual plane. Each surface must be small enough to effectively approximate the surface fluctuation. The spatial geometry parameters of a small surface element are determined by its central position vector and normal vector. The open-pit mine in this case study was subject to daily blasting operations which also shook the command center (building in Figure 6). Slope stability in the lower part of the command center is the primary target of mine safety applications.

**Figure 7:**
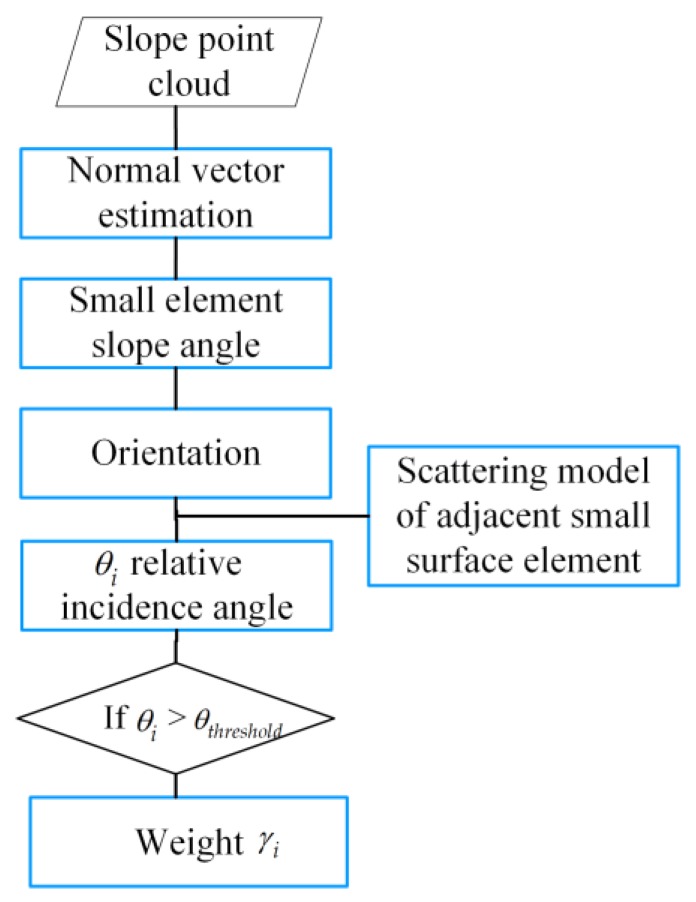
Assignment weights according to point cloud incident angle geometric-scattering model.

An algorithm flow was established (Figure 7) to explicitly show how to determine the weights assigned to the point cloud by the radar pixel based on the geometric-scattering model. K values need to be set according to the difference in resolution between the point cloud and the radar image. The normal vector can be computed using the method introduced by Hoppe et al. based on the directed distance function (signed short function) of the scattered point cloud the normal vector [19]. Assuming that the terrain point cloud plane is continuous after sampling, the most similar K adjacent points can be determined for each point p by the k-nearest neighbor classification method, which has been widely used, and as such there is no need to describe it in this paper. The local incident angle can be obtained quickly via Equation (4). The focal point coordinates $S(x_{1},y_{1},z_{1})$ and the phase center coordinates $A(x_{2},y_{2},z_{2})$ of the small surface element allow the local incident angle $\theta_{i}$ for each small surface element to be computed as:

(4) $$\theta_{i}=\arccos\frac{-\vec{n_{i}}\cdot \vec{SA_{i}}}{\left|{\vec{n}}_{i}||\vec{SA_{i}}\right|},$$

where ${\vec{n}}_{i}$ represents the normal vector of the i*th* point in the terrain cloud.

The steepness in the k-neighborhood of each point on the surface can be calculated with a differential operation. A smaller value indicates flatter terrain in the neighborhood and vice versa. The slope of the k-neighborhood at each point can be calculated as follows:

(5) $$\theta_{slope}=\arctan\sqrt{f_{x}^{2}+f_{y}^{2}},$$

where $\theta_{slope}$ is the radian value of the slope angle in the k neighborhood,$f_{x}$ is the change rate of elevation in the x direction, and $f_{y}$ is the change rate of elevation in the y direction. The direction vector of the small face element is calculated based on the slope of the k-neighborhood as:

(6) $$\theta_{aspect}=\arctan\sqrt{\frac{f_{y}}{f_{x}}},$$

where $\theta_{aspect}$ is the orientation of the k-neighborhood. The orientation represents the maximum change direction of the elevation change at any point in the terrain point cloud. The standard deviation of surface heights was used to evaluate the degree to which the surface fluctuates and to judge whether the k-nearest neighbor surface element scattering model can be used. Suppose a surface is in the x–y plane and the height of some point (x,y) above this plane is z(x,y). Assuming that the center of the surface is at the origin, then the average height of the surface can be calculated by Equations (7) and (8).

(7) $$\bar{z}=\frac{1}{L_{x}L_{y}}\int_{-L_{x}/2}^{L_{x}/2}\int_{-L_{y}/2}^{L_{y}/2}z(x,y)dxdy,$$

The corresponding secondary moment is:

(8) $$\bar{z^{2}}=\frac{1}{L_{x}L_{y}}\int_{-L_{x}/2}^{L_{x}/2}\int_{-L_{y}/2}^{L_{y}/2}z^{2}(x,y)dxdy,$$

The standard deviation of the surface heights σ

(9) $$\sigma ={\left(\bar{z^{2}}-{\bar{z}}^{2}\right)}^{\frac{1}{2}},$$

The Fraunhofer criterion of the antenna’s far-field condition is also useful here. If the maximum phase difference between the center and edge of the antenna and the receiving point is greater than or equal to π/8, it can serve as the criterion of the scattering model.

(10) $$\sigma \geq \frac{\lambda }{32\cos\theta_{i}},$$

where λ is the wavelength. The direction vector of orientation can replace the normal vector of k-nearest neighbors in Equation (4) to calculate the point’s k-neighborhood relative to the radar incidence angle:

(11) $$\theta_{i}=\arccos\frac{-n_{aspect}\cdot \vec{SA}}{\left|n_{aspect}||\vec{SA}\right|},$$

where $\vec{SA}$ is explicated in Figure 5a. The equation of the upper hemisphere using Lambert’s law is the weight assigned to the cosine graph according to the distribution of Equation (12):

(12) $$\gamma (\theta_{i})=\gamma (0)\cos\theta_{i},$$

where γ(0) is the initial value of the weight can be determined by other methods with distinct geometric features. The scattering observed here is caused by the projected area. The spheres are independent of each other and separated far enough so that they do not overlap in any way. Therefore, the re-radiation directivity diagram of the isotropic spheres remains unchanged. 

The next section shows how to use the proposed process with simulated data, and then verifies the proposed method with actual data. 

## 4. Results

**Figure 8:**
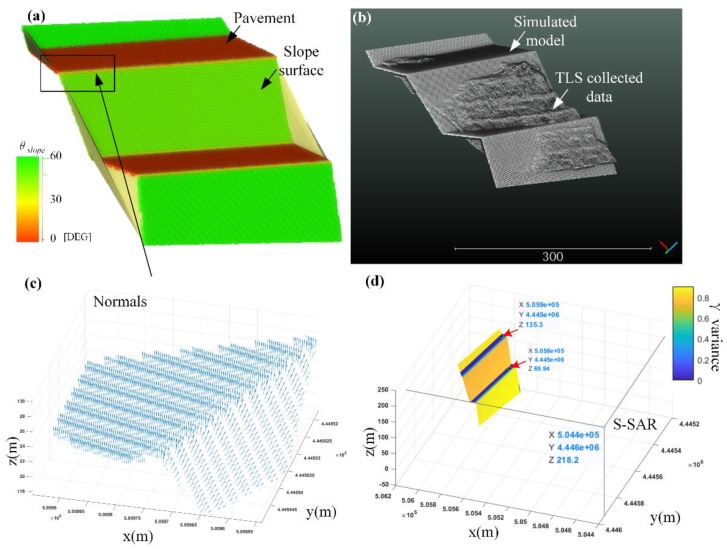
(**a**) Surface color of the road-slope model indicating $\theta_{slope}$ variance extracted by Equation (5). (**b**) Geocoded model with the actual collected terrain point cloud to a local coordinate system via linear transformation. (**c**) Normal vectors of the pavement and slope of the rectangle. (**d**) $\gamma (\theta_{i})$ the variance of each point. The deep blue color area is the pavement. In this case, they have a bad incident angle.

A slope-pavement model was established (Figure 8a). The step model consists of slope and pavement components. The deformable regions were simulated across the road and slope on a deformation image based on the simulated monitored slope. The nearest-neighbor interpolation method and the proposed method were used separately for mapping to observe whether the projected deformation was consistent with the shape of the step. The model has been geocoded with the target area based on block stretching and linear transformation (Figure 8b).

- Normal $\vec{n_{i}}$: Normal vector computing is integrated into many point cloud processing software and C++ library, for example: Cloud compare, MeshLab, and point cloud library (PCL). We programmed with the PCL library and referenced the Cloud compare built-in minimum spanning tree method to extract normal vectors of the sampled step model. $\vec{n_{i}}$ is as shown in Figure 8c. The pavement and the slope can be differentiated by $\vec{n_{i}}$.
- Orientation: The orientation of each point $n_{aspect}$ in the model is easy to extract. As the step model coded, it has a certain horizontal coordinate system plane. The angle between $\vec{n_{i}}$ and horizontal plane normal can be treated as an orientation vector $n_{aspect}$, and the $\theta_{aspect}$ of each point in Equation (6) is identified.
- Line-of-sight (LOS): LOS is the vector that connects each point to radar Station *S*.
- Relative incident angle $\theta_{i}$: $\theta_{i}$ can be determined by Equation (11).
- Weight $\gamma (\theta_{i})$: $\gamma (\theta_{i})$ is the normalization of $\cos(\theta_{i})$. The result is shown in Figure 8d.

One rather important thing to note, the simulated stepped model is more like a “smooth surface” consist of many particles. However, the target area in a natural scene is more like a “rough surface”. Figure 8d shows the weight difference of the stepped model. The pavement weight was much lower than the slope surface through SAR beam incidents in an overlooking perspective. The weight of pavement only means the incident angle is not good, which may lead to a disappearance of deformation in the geometric mapping process. The topographically rugged surface of the pavement will not be affected by the deformation disappearance. 

**Figure 9:**
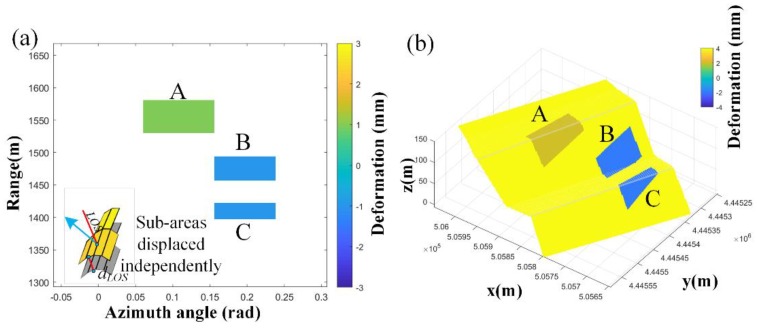
(**a**) Simulated sub-areas with small deformation 2D deformation map. (**b**) Geometrical mapping 3D result of deformation in (**a**) using a nearest-neighbor interpolation method.

Sub-area deformation positioning: The mapping relation table of the 3D point cloud and radar image was established via geometric mapping. Several subregions were identified in the diagram that are composed of groups of pixels that appear as blocks on the surface. As shown in Figure 9a, these blocks have a global displacement along the LOS direction; this simulates the displacement detected by the radar rather than changes in the 3D model. The simulation results can then be mapped to a 3D space as shown in Figure 9b. This case features sub-area overall deformation. The GB-InSAR image as-collected from the open-pit mine was used to match the real terrain point cloud and simulated terrain point cloud using the method described in Section 2. Several sub-areas that cover the pavement and slope surface were then created as shown in Figure 9a–c were the index of the sub-area. However, in the real world, the deformation of the road surface and slope surface is always different, so simulation case 1 only shows the spatial positioning of each sub-area.

**Figure 10:**
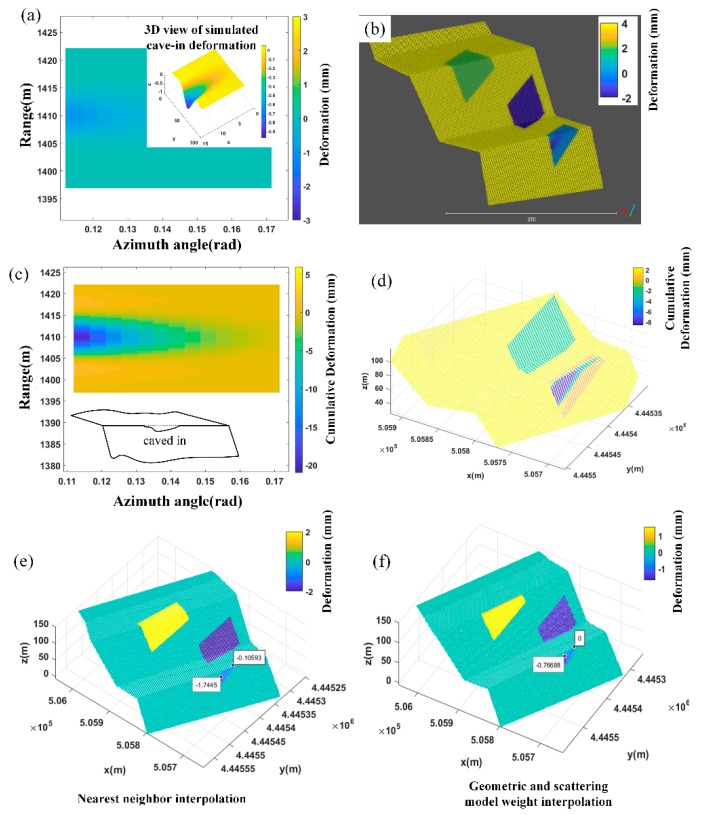
(**a**) 2D deformation diagram shows a slight caving-in phenomenon. (**b**) The nearest-neighbor method creates a 3D result which has no obvious discrimination between slope and road surfaces. (**c**) Simulated deformation diagram of Region D after accumulation. (**d**) Accumulated 3D matching produces obvious banding. A similar phenomenon occurs in adjacent regions to D. (**e**) Nearest-neighbor method result. (**f**) The result generated by the geometric and scattering weight model, where deformation results distinguish road surface from the slope surface. The pavement deformation disappears because the incident angle is not good. If the pavement surface is rugged as shown in Figure 6, the pavement deformation would be reserved.

Continuous deformation generated through simulation in Region C: This was a simulation of a slope surface collapse, where the flow of water in the slope was caused by internal empty surface sag. The deformation field was simulated via a quadratic function model, as shown in Figure 10a. At this point, the lower part of the slope moved down relative to the radar resulting in a negative displacement relative to the radar. This was then projected into 3D space using the nearest-neighbor method (Figure 10b). If the area produces persistent, identical depressions and is detected by GB-InSAR, then the subsequent deformation image is the same as the image shown in Figure 10c. Then the deformation values of each pixel can be accumulated to form the image. At this point, there is a gradual and non-linear deterioration of the slope. If a pixel is across the slope and road surface, the assignment of the point cloud within the pixel set should be distinguishable. Scattering model analysis can solve this problem. If the radar wave incident angle of the road is poor but that of the slope is acceptable, then the nearest-neighbor method results of the road surface and slope are the same. The proposed method makes the road result more distinguishable by reducing its weight and strengthening slope results. Based on the above assumptions, the normal vector of each point was calculated followed by the slope angle of the small surface element adjacent to each point. 

**Figure 11:**
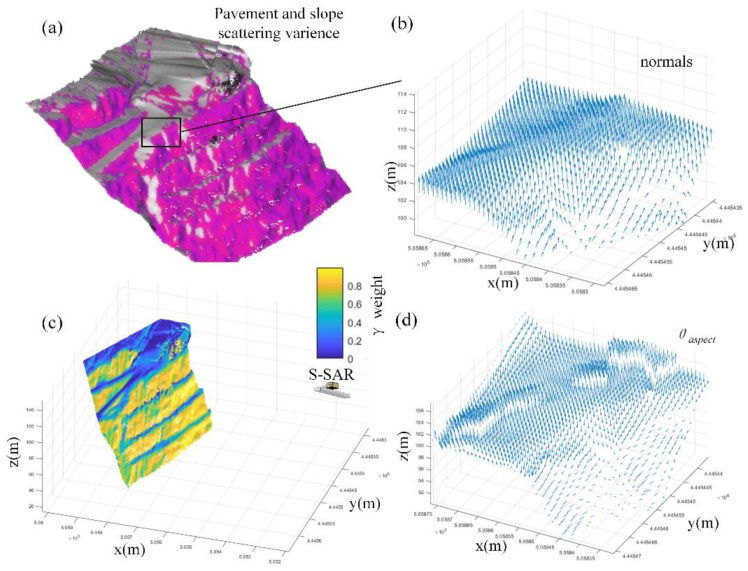
(**a**) Radar local incident angle and (**b**) normalized results, local zoom in Figure 11a. The arrow represents the direction of the incident vector (Equation (11)). (**c**) Weight varies when the scattering model changes. (**d**) The arrow represents the direction of local most likely deformed by gravitation or explosion fluctuation.

**Figure 12:**
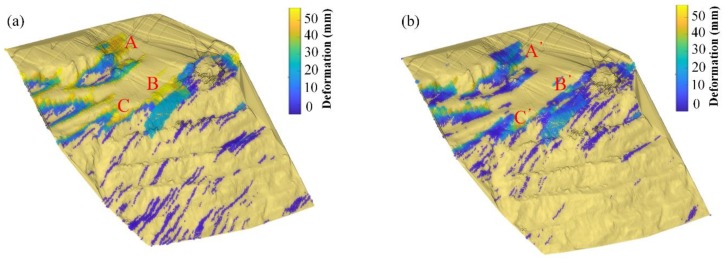
The cumulative deformation map from GMT+8 13:03 10 October 2018 to 17:23 was used in the mapping experiment. Three sub-areas were also selected. (**a**) Nearest-neighbor interpolation mapping result. (**b**) Paper method mapping result.

Target area real application: Firstly, the pavement and slope scattering variance can be extracted by the S-SAR microwave incident angle and is colored as shown in Figure 11a. The bad incident areas have no color. The example figure of normal and calculated $\theta_{aspect}$ is shown in Figure 11b,d. The $\theta_{aspect}$ shows that the sub-area would most probably move towards a given direction when affected by gravitation. However, when they are affected by other kinds of outside forces, the model should couple with the force analysis. Since the target area is a permanent slope which is only fluctuated by daily mining explosion, it can be treated as deforms along $n_{aspect}$ vector. The weight $\gamma (\theta_{i})$ variance is shown in Figure 11c. The areas with bad incident angles are shown as deep blue and brighter yellow areas are areas with good incident angles. As the road surface height went lower, the incident situation got better. The dark color of each road edge is the soil retaining wall which is to protect trucks from falling. The wall area weights also vary by incident angle changes. The nearest-neighbor mapping result and paper method result is as shown in Figure 12. 

**Figure 13:**
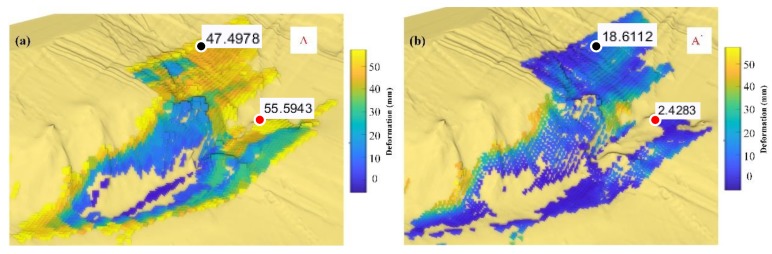
The deformation of the transition zone is removed to a certain extent by the model weighting method of the paper. These edge boundary anomalies, however, are often marginal areas with poor data quality, but are affected by strong coherence scattering points. (**a**) Nearest-neighbor interpolation mapping result of sub-area A. (**b**) Paper method mapping result of sub-area A’.

Three sub-areas A, A’, B, B’, C. C’ were selected, corresponding to the simulated step model sub-areas. Area A is as shown in Figure 13a, the bright yellow boundary shows a great deformation, with the highest reach near 56 mm, which is quite large. The situation is quite similar to the controlled field test from in our previous work [1], which are abnormal boundaries caused by the persistent scattering points that affect the neighborhood area. The area itself is not in a good coherence situation and the phase error is significant. Then, the deformation extracted in these boundary areas is also abnormal. In the A’ area, as is shown in Figure 13b, the situation is relieved, especially for the pavement areas: For the red point, from 55.5943 mm to 2.4283 mm; for the black point from 47.4978 mm to 18.6112 mm. For the slope area, the real deformation still exists, a geologist can introduce their analysis method for further destruction deduction. 

**Figure 14:**
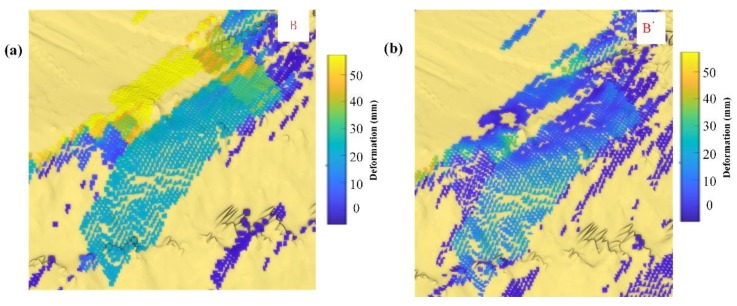
Multiple terrain surface deformed points may locate in one radar pixel spatial resolution element which crosses pavement and slope. The local layover distortion is hard to identify without geometric and scattering information. (**a**) Nearest-neighbor interpolation mapping result of sub-area B. (**b**) Paper method mapping result of sub-area B’.

GB-InSAR monitoring is flexible, and monitoring geometry should be well designed to balance between layover distortion and shadow. The local incident angle and K-nearest neighbor patches aspect angle in the geometric and scattering model may help the layover distortion analysis (the case is shown in Figure 14). The layover distortion would mostly happen when the SAR beam incident angle is near perpendicular. The area B and B’ show the same radar spatial resolution element. The B area shows no difference when across the road and slope. The sub-area deforms about 20 mm during the experiment. However, for the paper method, the B’ showed a difference as the incident angle changes. The road and slope were differentiated, and the inside small geo-elements deform according to their local shape. The result would help small area (less < 200 m^2^) slide analysis, which often happens in open-pit mines. 

**Figure 15:**
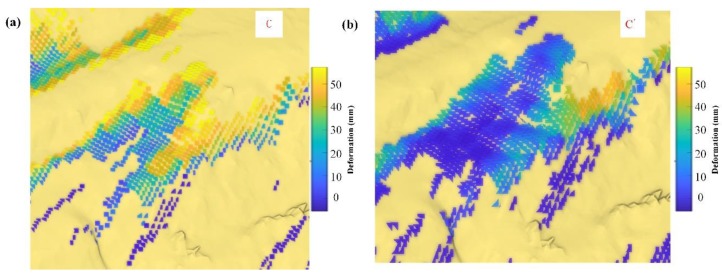
A deformation region with large local undulations. (**a**) Nearest-neighbor interpolation mapping result of sub-area C. (**b**) Paper method mapping result of sub-area C’.

As shown in Figure 15, the areas C and C’ show an area with an undulated surface. The case is quite similar to the road and slope crossing area. These areas are commonly seen in an open pit where roads go two directions, or the roads are a regional rotary; these are often unstable when trucks pass. The abnormal boundary was relieved by the proposed method in the paper. The nearest-neighbor method result is confused by abnormal deformation and the real deformed area is concealed.

## 5. Discussion

This section concentrates on the following issues:

- How the weight influences the deformation mapping result.
- How can we get closer to the real deformation.

**Figure 16:**
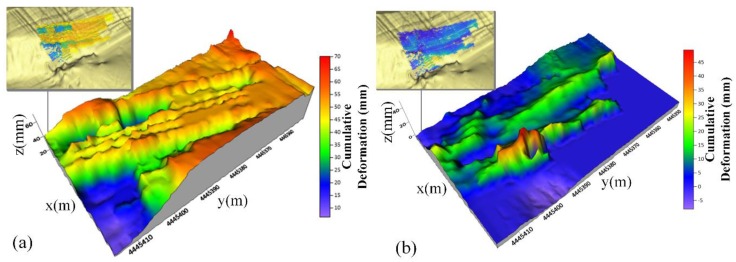
The deformation in one radar pixel generated by two methods. (**a**) Nearest-neighbor interpolation deformation result. The whole pixel may equal one vital scattering target. (**b**) Deformation result using the geometric and scattering model. The terrain model point cloud may equal several distributed targets.

In a strict sense, the weight calculated based on Equation (12) in the paper may not conform to the scattering differences of local areas in any natural scene. However, it is widely used in SAR image simulation. It comes from the hypothesis that the surface is a collection of “small spherical” scatterers. Some scattering model is simulated based on cosθ function and a lot of researchers adopt the $\cos^{2}\theta$ function to simulate the scattering difference. In addition, if possible, we need to design our own compromise function to accommodate the local geological condition. The factor will affect deformation mapping. For example, the result in Figure 13 shows sudden changes from 40–50 mm to 20–30 mm. The situation may confuse us if the mapping result is correct or near the real deformation. Figure 16a,b is the one radar pixel interpolated deformation surf plot of A and A’ (Figure 13). The figure would help to understand the influences when using a geometric-scattering model.

According to Figure 16, the following understanding can be concluded.

- The GB-InSAR image pixel contains several targets. Due to the limited spatial resolution, these targets can be equivalent to one vital “scattering target”. The terrain point cloud model spatial resolution is higher than radar. The local area with a good incident angle in the radar pixel plays a major role in forming "sub-targets", and the shape variables are distributed on these distributed sub-targets. In this study, the deformation on the road surface is not eliminated, and their deformation is concentrated on the distributed sub-targets with good incident angles on the road surface.
- If the precise deformation in the radar pixel is known through the ground control points or global navigation satellite system (GNSS) and other high time resolution measuring instruments, a more accurate model of mapping can be established. The optimizing objective function the least square solution of the temporal sequence deformation of each sub-target and the deformation function of the radar pixel.

From the perspective of geotechnical application, the difference between slope and road surface is highlighted, and the instability can be analyzed from a macroscopic perspective. Moreover, the model proposed in this paper can be integrated with the existing classical methods, rather than abandoning the previous methods. Fusion analysis is not the focus of this paper, and it will be further studied in the future.

## 6. Conclusions

In this study, geometric and scattering models were established to solve the “one-to-many” problem and pavement, slope differentiation. A scattering and geometric weighting model was proposed during the interpolation process of mapping. A simulation case and a target area field test were conducted to test the method and compared it with the nearest-neighbor interpolation method. The following problems were resolved:

- The pavement and slope surface deformation were differentiated.
- The parameters can be adjusted to avoid band-like phenomena in the experiment.
- The abnormal deformed boundaries were relieved to a certain extent.

The geometric and scattering models are not without defects. In the future, more adaptable models should be introduced according to regional conditions of the slope under analysis so that the deformation measured by GB-InSAR can be best integrated with other geographical models. This will allow geologists to analyze the safety of unstable slopes.
